# Attenuation of indirect markers of eccentric exercise-induced muscle damage by curcumin

**DOI:** 10.1007/s00421-015-3170-4

**Published:** 2015-04-29

**Authors:** Yoko Tanabe, Seiji Maeda, Nobuhiko Akazawa, Asako Zempo-Miyaki, Youngju Choi, Song-Gyu Ra, Atsushi Imaizumi, Yoshihiko Otsuka, Kazunori Nosaka

**Affiliations:** Graduate School of Comprehensive Human Science, University of Tsukuba, Tsukuba, Ibaraki Japan; Faculty of Health and Sport Sciences, University of Tsukuba, 1-1-1 Tennodai, Tsukuba, Ibaraki 305-8574 Japan; Faculty of Medicine, University of Tsukuba, Tsukuba, Ibaraki Japan; Theravalues Corporation, Tokyo, Japan; Centre for Exercise and Sports Science Research, Edith Cowan University, Joondalup, WA Australia

**Keywords:** Polyphenolic, Elbow flexors, Muscle strength, Muscle soreness, Supplement, Creatine kinase

## Abstract

**Purpose:**

Polyphenolic curcumin is known to have potent anti-inflammatory effects; thus the present study investigated the hypothesis that curcumin ingestion would attenuate muscle damage after eccentric exercise.

**Methods:**

Fourteen untrained young men (24 ± 1 years) performed 50 maximal isokinetic (120°/s) eccentric contractions of the elbow flexors of one arm on an isokinetic dynamometer and the same exercise with the other arm 4 weeks later. They took 150 mg of curcumin (theracurmin) or placebo (starch) orally before and 12 h after each eccentric exercise bout in a randomised, crossover design. Maximal voluntary contraction (MVC) torque of the elbow flexors, range of motion of the elbow joint, upper-arm circumference, muscle soreness, serum creatine kinase (CK) activity, and plasma interleukin-6 (IL-6) and tumor necrosis factor-α (TNF-α) concentration were measured before, immediately after, and 24, 48, 72 and 96 h after each eccentric exercise. Changes in these variables over time were compared between curcumin and placebo conditions by two-way repeated measures ANOVA.

**Results:**

MVC torque decreased smaller and recovered faster (e.g., 4 days post-exercise: −31 ± 13 % vs. −15 ± 15 %), and peak serum CK activity was smaller (peak: 7684 ± 8959 IU/L vs. 3398 ± 3562 IU/L) for curcumin than placebo condition (*P* < 0.05). However, no significant differences between conditions were evident for other variables, and no significant changes in IL-6 and TNF-α were evident after exercise.

**Conclusion:**

It is concluded that theracurmin ingestion attenuates some aspects of muscle damage such as MVC loss and CK activity increase.

## Introduction

Unaccustomed exercise, especially that consists of high-intensity and/or large number of eccentric (lengthening) contractions, induces muscle damage (Clarkson et al. 1992). Eccentric exercise-induced muscle damage is indicated by prolonged decreases in muscle strength and range of motion (ROM), swelling, delayed onset muscle soreness (DOMS), and increased muscle proteins in the blood such as creatine kinase (CK) activity (Warren et al. 1999). Muscle damage initiates inflammatory responses, resulting in secondary muscle damage (Tiidus 1998). The impairment of muscle function caused by damage and subsequent inflammatory responses could reduce the ability to perform daily activities and athletic performance. Thus, it is important to prevent or minimise muscle damage after exercise by attenuating inflammatory responses. One of many measures for this purpose could be an oral intake of anti-inflammatory substances (Connolly et al. 2003).

Several studies have shown that some anti-inflammatory substance supplementations are beneficial for attenuating eccentric exercise-induced muscle damage (Connolly et al. 2006; Howatson et al. 2010; Trombold et al. 2010), but other did not find such effect (O’Fallon et al. 2012). These inconsistent results may, in part, be due to the differences in substances, exercise model and experimental design among studies. It has been reported that tart cherry juice consumption enhanced recovery of muscle strength and attenuated interleukin-6 (IL-6) and C-reactive protein responses after marathon running (Howatson et al. 2010) and prolonged upper arm exercise (Connolly et al. 2006). Other study showed that taking polyphenol ellagitannins from pomegranate extract before and after high-intensity elbow flexor eccentric exercise attenuated muscle strength loss by 10 % (Trombold et al. 2010). In contrast, O’Fallon et al. (2012) reported that ingestion of a polyphenol flavonoid from quercetin before and after exercise did not affect changes in maximal voluntary contraction (MVC) strength, ROM, serum CK activity, IL-6 and tumor necrosis factor-α (TNF-α) and DOMS after elbow flexor eccentric contractions.

Curcumin, extracted from turmeric, is a natural polyphenolic substance. It has been demonstrated that curcumin has anti-inflammatory effect by downregulating the nuclear factor-kappa B (NF-kB), thereby suppressing the expression of IL-6 and TNF-α (Cho et al. 2007; Aggarwal et al. 2006). The anti-inflammatory effects of curcumin have been used in the treatment of rheumatoid arthritis, inflammatory bowel disease, multiple sclerosis and diabetes (Sethi et al. 2009). Usharani et al. (2008) demonstrated that 600 mg of daily curcumin intake for 8 weeks reduced approximately 60 % of plasma IL-6 and TNF-α concentrations in diabetes mellitus patients. Polyphenol supplementation of curcumin has been reported to blunt IL-6 and TNF-α increases in mice muscle after downhill running (Davis et al. 2007). Kawanishi et al. (2013) have recently reported that oral curcumin ingestion (3 mg) suppresses oxidative stress, reducing hydrogen peroxide in skeletal muscle following downhill running-induced muscle damage in mice. Thus, it is possible that curcumin supplementation is effective for attenuating eccentric exercise-induced muscle damage in humans. However, to the best of our knowledge, no previous human study has examined the effects of curcumin ingestion on markers of muscle damage after eccentric exercise.

The purpose of this study, therefore, was to examine the acute effect of curcumin intake before and 12 h post-exercise on changes in indirect markers of muscle damage and inflammation after eccentric elbow flexors exercise. It was hypothesised that curcumin ingestion would attenuate the changes in indirect muscle damage markers and inflammatory markers in the blood after eccentric exercise when compared with placebo condition.

## Methods

### Subjects

The present study recruited 14 healthy, untrained young men who had not been involved in any regular resistance training for at least 1 year before this study. Their average ± SD age, height and body mass were 23.5 ± 2.3 years, 172.1 ± 7.5 cm and 65.2 ± 11.3 kg, respectively. The sample size was estimated on the assumption that the curcumin ingestion could attenuate the decrease in muscle strength in recovery days by at least 25 %, if it was effective. On the basis of the power of 0.80 and alpha level of 0.05, a sample size of 10 was found to be necessary based on the data of a previous study (Evans et al. 2002) in which a similar eccentric exercise to that of the present study was performed. Subjects maintained their normal food intake and lifestyle habits, but abstained from strenuous physical activities, and did not take anti-inflammatory drugs during the study period. All subjects gave their written informed consent to participate in the study, and all procedures were reviewed and approved by the ethical committee of the University of Tsukuba.

### Experimental design

This study used a crossover design in which one arm was used for curcumin condition, and the other arm was used for placebo condition in a randomised, single-blinded fashion. This design requires a smaller number of participants, and it has been reported that a within-subject (contralateral arm) design is an alternative to a between-subject design if the order of exercise is counterbalanced by arm dominance (Newton et al. 2013).

Each arm performed a bout of eccentric exercise of the elbow flexors separated by 4 weeks, and the use of dominant-nondominant arms and the order of the conditions were counterbalanced among the subjects. Dependent variables consisted of MVC torque of the elbow flexors, ROM of the elbow joint, upper-arm circumference, muscle soreness, serum CK activity, and plasma IL-6 and TNF-α concentrations. Changes in these variables before, immediately after and 24, 48, 72 and 96 h after the exercise were compared between the curcumin and placebo conditions.

### Curcumin and placebo

We developed theracurmin (Theravalues Corporation, Japan) using a microparticulation and surface processing technique, and theracurmin has been shown to result in much higher plasma concentration and bioavailability after intake when compared with conventional curcumin powder (Sasaki et al. 2011). As a placebo, starch was used, which was contained in an identical capsule as that of the curcumin, so the subjects could not distinguish between them. Six capsules of 25 mg of curcumin or placebo were administered 1 hour before (150 mg) and 12 h after exercise (150 mg). It has been reported that post-exercise inflammation starts to present around 4–12 h after eccentric exercise, and further develops in 24–48 h after exercise (Paulsen et al. 2012; Davis et al. 2007). Considering the time taken for the curcumin to get into the blood stream, and its remaining time in the blood, it was assumed that the ingestion at 1 h before exercise could attenuate the initial inflammation, and that at 12 h after exercise could attenuate the development of inflammation before 24 h post-exercise. The amount (150 mg) has been shown to be safe for one time dose (Kanai et al. 2012). However, it had not been known before this study how much of curcumin would be effective to prevent muscle damage in humans, so we assumed that a significant difference between the curcumin and placebo conditions would be shown by the dose (150 mg before and 12 h after exercise, 300 mg/day), if any positive effect of curcumin ingestion on muscle damage existed.

### Eccentric exercise

All subjects performed two bouts of eccentric exercise of the elbow flexors on a BIODEX dynamometer (BIODEX System 3, USA), using one arm for each bout separated by 4 weeks. Each subject was seated on the dynamometer chair with the arm being supported by padded armrest secured at 45° shoulder flexion and the waist and chest being stabilised with straps. The exercise consisted of 50 maximal eccentric contractions of the elbow flexors at an angular velocity 120°/s, and the range of motion was from 130° and to 10° elbow flexion, where a fully extended elbow joint angle was considered to be 0°, thus each contraction time was 1 s (Evans et al. 2002). The exercise limb was passively returned to the initial position at 10°/s, creating a 12-s rest between contractions. The subjects were instructed to contract the elbow flexors maximally to resist the elbow extending action of the dynamometer for the whole range of motion. Total work and peak torque were calculated using the BIODEX System software.

### Muscle damage markers

Several indirect markers of muscle damage, which were often used in previous studies (Nosaka et al. 2002a, b), were measured before, immediately after, and 24, 48, 72 and 96 h after each exercise bout. The details of the markers are shown below. The test–retest reliability of each measure was determined using two baseline measurements performed before exercise by an interclass correlation coefficient (*R*) and coefficient of variation (CV). The *R* and CV (shown in parentheses) values for MVC torque, ROM and upper-arm circumference were 0.99 (2.9 %), 0.97 (1.1 %) and 1.00 (0.2 %), respectively. The muscle soreness was not assessed for the reliability, because all participants recorded DOMS scores as zero for palpation during the familiarisation sessions. The intra-assay CV was 5.3 % for CK activity, 3.2 % for IL-6 and 10.0 % for TNF-α in a pilot study using five men.

#### MVC torque

MVC torque of the exercised elbow flexors was measured using the isokinetic dynamometer in the same positioning as that of the eccentric exercise, and the elbow joint angle was set at 90°. Three 5-s maximal isometric contractions were performed with a 30-s rest between trials, and the highest value of the three trials was used for further analysis (Nosaka et al. 2002a).

#### ROM

According to the protocol of the previous study (Nosaka et al. 2006), each subject was asked to actively extend the elbow joint maximally (extended elbow joint angle) and to touch shoulder of the same side with the hand (flexed elbow joint angle). The ROM was determined as the difference between the two elbow joint angles. Each angle was measured by a goniometer three times, and the mean of the three measures was used for the calculation of ROM.

#### Upper-arm circumference

To assess muscle swelling, upper-arm circumference was assessed at the mid-belly of biceps brachii muscle (60 % distal to the length between the acromial process and epicondylus lateralis humeri) using a standard tape measure (Evans et al. 2002). The measurements were taken three times, and mean value of the three measurements was used for further analysis.

#### Muscle soreness

Muscle soreness upon palpation of the upper-arm and passively extending the elbow joint was quantified by a visual analog scale (VAS) that had a 100-mm line with “no pain” on one end and “extremely sore” on the other end (Chen and Nosaka 2006). The subjects were instructed to sit with arm relaxed, and the investigator palpated over the biceps brachii, and extended the elbow joint maximally. The same investigator performed all tests and the pressure of palpation was kept as constant as possible between subjects and between days.

#### Blood sampling and analyses

Blood was taken from the antecubital vein by a standard venipuncture technique. To obtain serum for CK activity analysis, 9 ml blood sample was collected using commercially produced vacuum-sealed serum collection tube (SEKISUI Medical Company Limited, Japan). To obtain the plasma for curcumin, IL-6, and TNF-α analyses, 5 ml blood sample was collected using a tube containing ethlenediaminetetraacetic acid (Terumo corporation, Japan). These were centrifuged at 3000 rpm for 15 min at 4 °C, and the serum and plasma samples were stored at −80 °C until analysis. Plasma curcumin concentration was measured by a HPLC–MS/MS system consisting of a Prominence micro-C system (Shimadzu, Kyoto, Japan) and an API 3200 tandem mass spectrometer (Applied Biosystems CA, USA). Serum CK activity was measured using a test kit (L-type CK, Wako Pure Chemical Industries, Japan) by a commercial laboratory. The normal reference range for male adults with this method is 48–259 IU/L. The concentrations of plasma IL-6 and TNF-α were determined using a commercial enzyme-linked immunosorbent assay kit (Quantikine Human IL-6, TNF-α R & D system Inc. Minneapolis, USA).

### Statistical analyses

Changes in the dependent variables over time were compared between the curcumin and placebo conditions by a two-way repeated-measures ANOVA. When the ANOVA showed a significant interaction effect, a Tukey’s post hoc test was used to locate differences between conditions. A Tukey’s post hoc test was also used to compare the pre-exercise and post-exercise values for each bout separately, when a significant time effect was found by the ANOVA. Inter-group comparison for the total work and mean eccentric peak torque during the exercise were made by a paired *t* test. Since a Kolmogorov-Smirov test showed that serum CK activity data was not normally distributed, the peak serum CK activity was compared between the condition using a non-parametric Wilcoxon’s rank test. The level of statistical significance was set at *P* < 0.05. All data are shown as mean ± standard deviation (SD).

## Results

### Plasma curcumin concentration

**Fig. 1:**
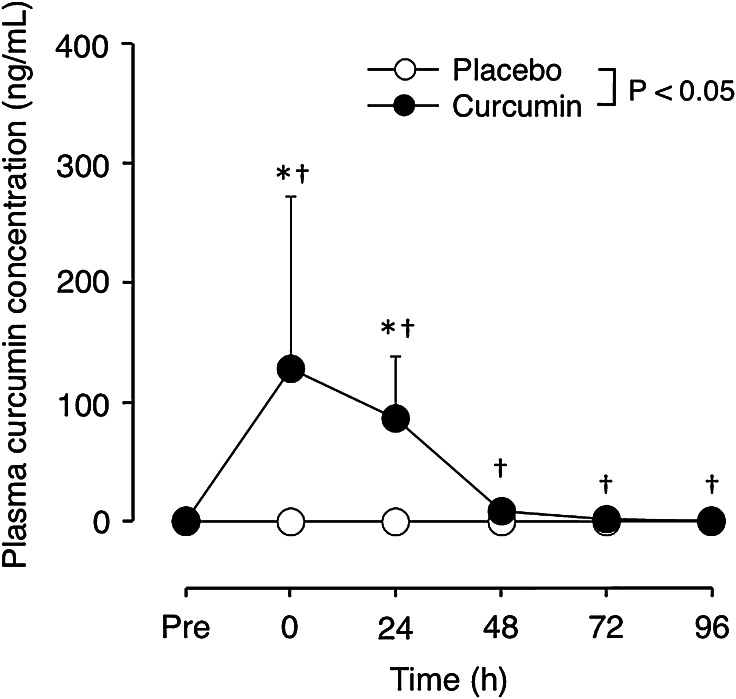
Changes (mean ± SD) in plasma curcumin concentration from the baseline (Pre), immediately (0) and 24–96 h after eccentric exercise of the elbow flexors for the curcumin and placebo supplementation conditions. *P < 0.05 vs. Pre; ^†^ *P* < 0.05 vs. placebo; *P* < 0.05: a significant interaction effect

There was no difference in plasma curcumin concentration at baseline between curcumin and placebo conditions, but a significant interaction effect was evident for the changes after exercise (Fig. 1). Plasma curcumin concentration increased from baseline (0.04 ± 0.07 ng/mL) to 127.7 ± 144.6, 85.7 ± 51.6, 8.6 ± 5.5, 2.2 ± 1.4 and 0.9 ± 0.6 ng/mL at 0, 24, 48, 72 and 96 h after exercise, respectively, for the curcumin condition. However, in the placebo condition, no significant changes in plasma curcumin concentration were observed from the baseline (0.01 ± 0.05 ng/mL) at any time points after exercise (0.02 ± 0.05, 0.02 ± 0.04, 0.03 ± 0.08, 0.03 ± 0.04 and 0.01 ± 0.02 ng/mL at 0, 24, 48, 72 and 96 h post-exercise, respectively).

### Exercise

The total work (2815 ± 788 J vs. 3011 ± 756 J, *P* = 0.178) and mean peak torque (40 ± 11 Nm vs. 41 ± 9 Nm, *P* = 0.597) during eccentric exercise did not differ between curcumin and placebo conditions.

### MVC torque

**Fig. 2:**
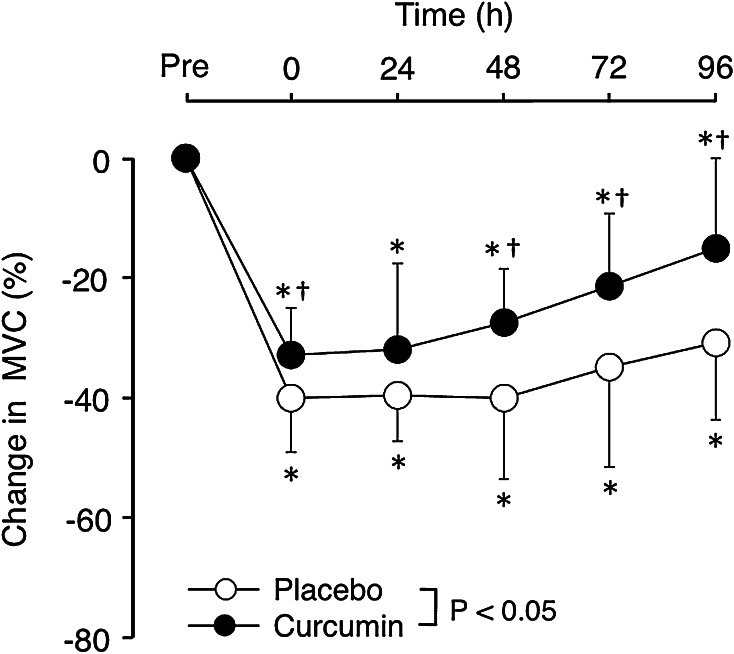
Normalised changes (mean ± SD) in maximal voluntary isometric contraction (MVC) torque from the baseline (Pre), at immediately (0) and 24–96 h after eccentric exercise of the elbow flexors for the curcumin and placebo supplementation conditions. *P < 0.05 vs. Pre; ^†^ *P* < 0.05 vs. placebo; *P* < 0.05: a significant interaction effect

The MVC torque before exercise was not significantly different between curcumin (52.5 ± 10.3 Nm) and placebo (55.1 ± 8.8 Nm) conditions. As shown in Fig. 2, a significant interaction effect was evident for the changes in MVC torque. The change in MVC from baseline was significantly smaller for the curcumin condition (33.0 ± 8.0 %) compared with placebo condition (40.0 ± 9.1 %) at immediately after exercise and also at 48–96 h after exercise by 13–16 %.

### ROM

**Fig. 3:**
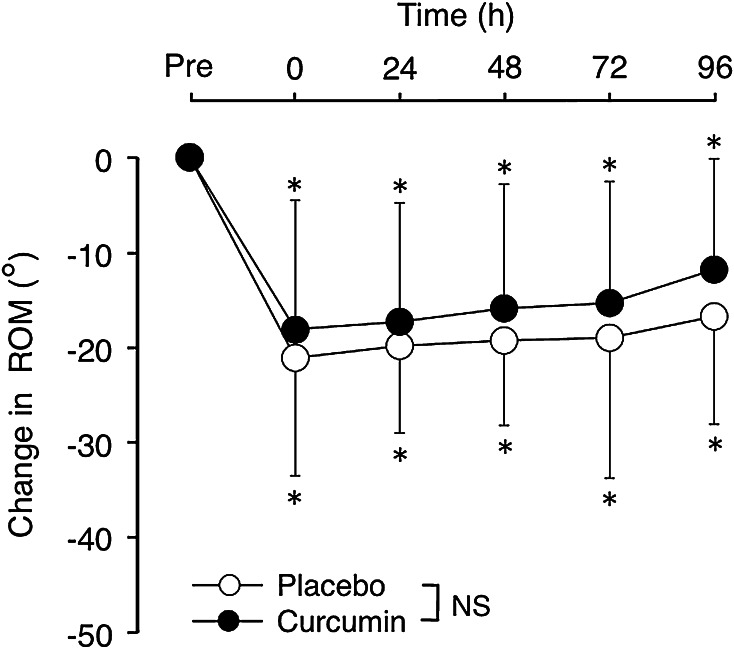
Absolute changes (mean ± SD) in range of motion (ROM) from the baseline (Pre) at immediately (0) and 24–96 h after eccentric exercise of the elbow flexors for the curcumin and placebo supplementation conditions. *P < 0.05 vs. Pre; *NS* no significant interaction effect

At the baseline, ROM was similar between curcumin (134° ± 4°) and placebo (133° ± 5°) conditions. After eccentric exercise, ROM significantly decreased at all measurement time points from the baseline, but no significant interaction effect was evident for the changes between conditions (Fig. 3).

### Upper-arm circumference

**Fig. 4:**
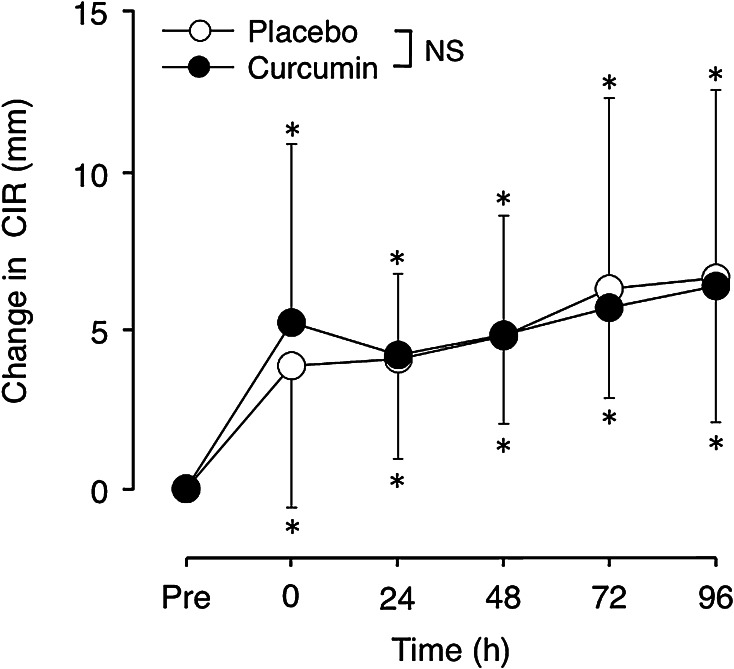
Absolute changes (mean ± SD) in upper-arm circumference (CIR) from the baseline (Pre) at immediately (0) and 24–96 h after eccentric exercise of the elbow flexors for the curcumin and placebo supplementation conditions. *P < 0.05 vs. Pre; *NS* no significant interaction effect

There was no significant difference in the baseline upper-arm circumference between curcumin (25.7 ± 2.5 cm) and placebo (25.9 ± 2.8 cm) conditions. Upper-arm circumference significantly increased after exercise by 3 % at 96 h post-exercise for both conditions, but no significant differences between conditions were found (Fig. 4).

### Muscle soreness

**Fig. 5:**
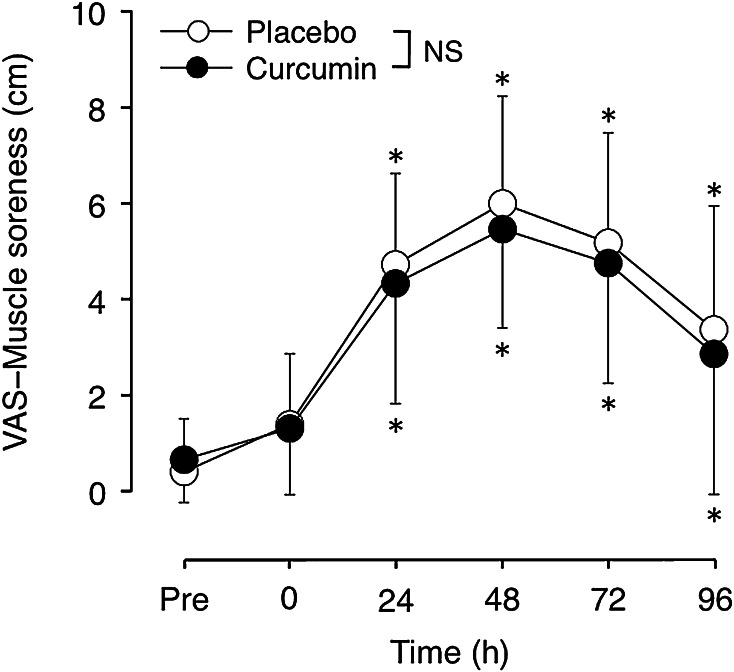
Muscle soreness upon palpation of biceps brachii (mean ± SD) assessed by a visual analog scale (10 cm) before (Pre), immediately after (0) and 24–96 h after eccentric exercise of the elbow flexors for the curcumin and placebo supplementation conditions. *P < 0.05 vs. Pre; *NS* no significant interaction effect

Significant increases in VAS were observed between 24 and 96 h after eccentric exercise for both conditions, and the peak VAS upon palpation was 5.5 ± 2.1 cm for curcumin and 6.0 ± 2.2 cm for placebo condition, but no significant interaction effect was evident (Fig. 5). This was also the case for the VAS with elbow joint extension, and the values were similar to those shown by the palpation.

### Serum CK activity

**Fig. 6:**
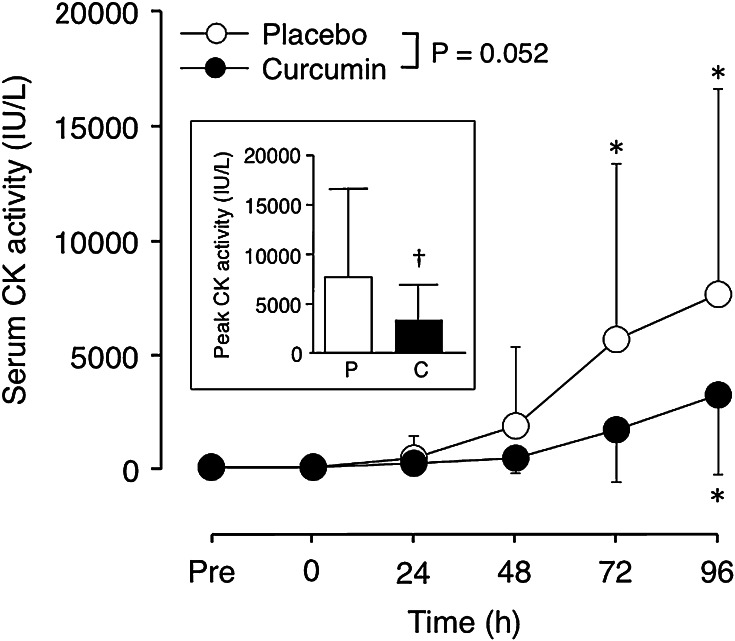
Changes (mean ± SD) in serum CK activity before (Pre), immediately after (0) and 24–96 h after eccentric exercise of the elbow flexors for the curcumin and placebo supplementation conditions. *P < 0.05 vs. Pre; An *inset*, peak serum CK activity for the curcumin (C) and placebo (P) conditions is shown; ^†^ *P* < 0.05 vs. placebo

Serum CK activity was within normal reference range prior to exercise without any significant difference between the curcumin (118 ± 49 IU/L) and control (115 ± 47 IU/L) conditions. Although the changes in serum CK activity after eccentric exercise were not statistically different between conditions (Fig. 6, *P* = 0.052), peak CK activity was significantly smaller for curcumin condition (3398 ± 3562 IU/L) than placebo condition (7684 ± 8959 IU/L).

### Plasma IL-6 and TNF-α concentration

**Fig. 7:**
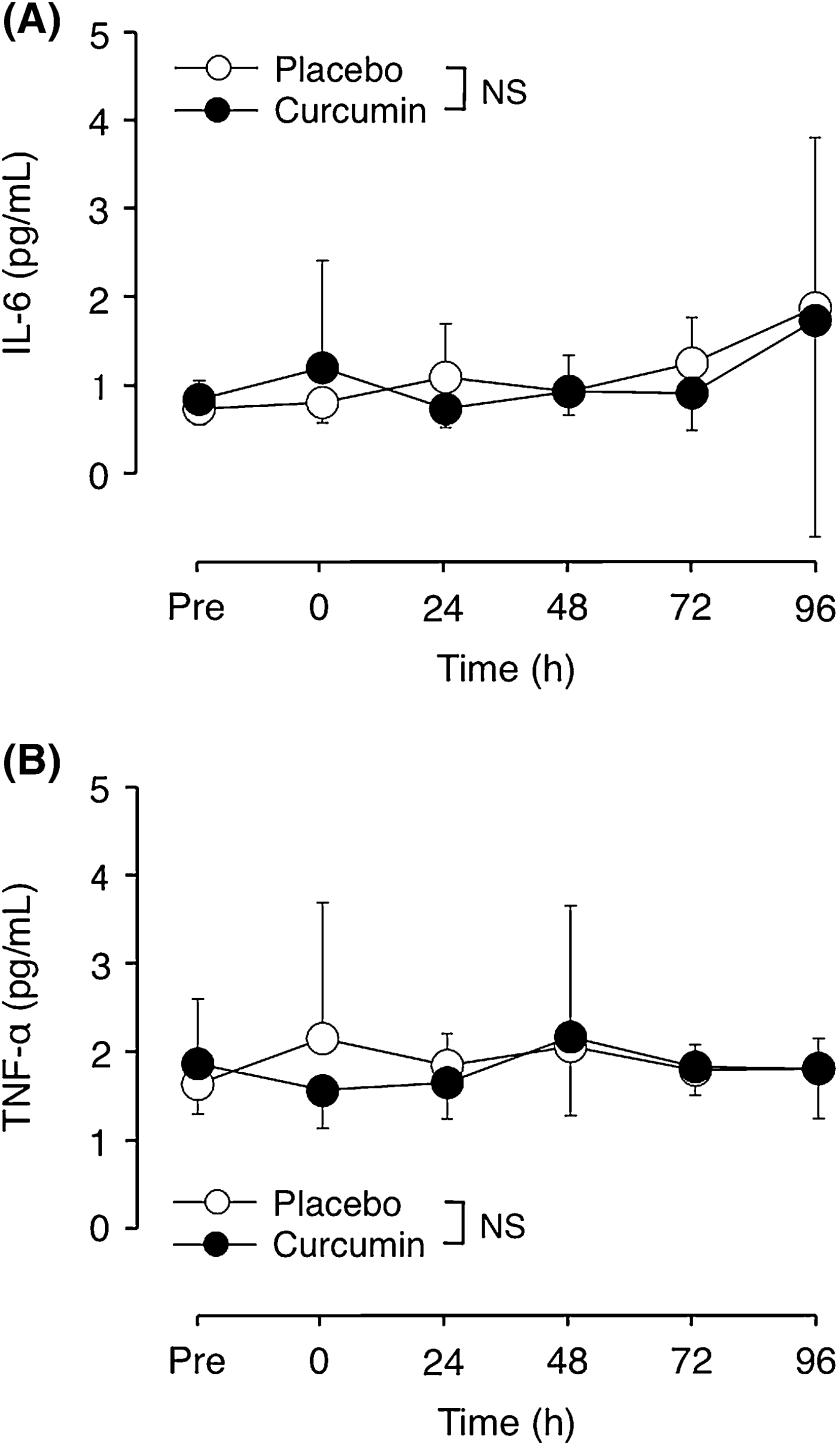
Changes (mean ± SD) in plasma IL-6 (**a**) and TNF-α (**b**) concentrations before (Pre), immediately after (0) and 24–96 h after eccentric exercise of the elbow flexors for the curcumin and placebo supplementation conditions. *NS* no significant interaction effect

Plasma IL-6 and TNF-α concentrations were not different between groups before exercise (IL-6 0.83 ± 0.22 vs. 0.73 ± 0.18 ng/mL, TNF-α 1.85 ± 0.74 vs. 1.63 ± 0.35 ng/mL, for curcumin and placebo, respectively). They did not change significantly after eccentric exercise, and no significant differences were found between curcumin and placebo conditions (Fig. 7).

## Discussion

This study investigated whether curcumin (theracurmin) ingestion at 1 h before and 12 h after eccentric exercise would attenuate muscle damage in healthy young men. The main findings of the study include that (1) plasma curcumin concentration elevated from the baseline after exercise only for curcumin condition (Fig. 1); (2) the magnitude of decrease in MVC torque was significantly smaller for curcumin than placebo condition (Fig. 2), (3) peak serum CK activity was significantly reduced in the curcumin than placebo condition (Fig. 6), but (4) no significant differences between the conditions were evident for other variables (Figs. 3, 4, 5, 7). These results did not fully support the hypothesis that curcumin ingestion would attenuate the changes in the muscle damage markers after eccentric exercise, but showed that the curcumin was effective for some aspects of muscle damage.

The present study used an arm-to-arm comparison model, and the test order of curcumin and placebo conditions, as well as the use of the dominant and nondominant arms, were counterbalanced among subjects. The total work and mean peak torques during eccentric exercise did not differ between conditions. Although a small (7 %) non-significant difference in the total work was observed between the conditions, the total work did not correlate with the magnitude of decrease in MVC torque. Thus, it seems reasonable to expect that changes in the dependent variables were similar between the conditions, if curcumin ingestion had not produced any effects. However, the magnitude of decline in MVC torque immediately after exercise for the curcumin condition (33 %) was significantly smaller than that of the placebo condition (40 %), and this difference was sustained in the recovery days (Fig. 2). These indicate that the curcumin ingestion immediately before and/or 12 h after exercise affected the changes in MVC torque.

Trombold et al. (2010) showed that daily ellagitannin intake reduced loss of isometric elbow flexion strength by 10 % at 48 and 72 h after exercise. Connolly et al. (2006) reported that cherry juice ingestion for 4 days before and for 4 days after exercise attenuated muscle strength loss by 20 %. These authors speculated that anti-inflammatory and/or anti-oxidant effect of the substances decreased secondary muscle damage responses such as increases in vascular permeability, neutrophil and free radical, attenuating muscle fibre disruption (Connolly et al. 2006; Trombold et al. 2010). It is important to note that increases in serum CK activity were also attenuated by the curcumin ingestion in the present study (Fig. 6). Increases in CK activity in the blood indicate disruption of plasma membrane of muscle fibres and are commonly used as a marker of muscle fibre damage (Clarkson et al. 1992). It was reported that morphological disruption of myofibrils was associated with the magnitude of decrease in muscle function (Raastad et al. 2010). In the present study, the smaller increases in serum CK activity in the curcumin condition compared with the placebo condition may suggest that myofibril damage was attenuated by curcumin ingestion. This might be associated with the smaller decreases in MVC torque for the curcumin condition. Moriyuki et al. (2010) reported that curcumin inhibited production of prostaglandin via suppression of cyclooxygenase-2 upregulation and NF-kB signals, implying that curcumin ingestion might decrease vascular permeability. It may be that curcumin stabilises plasma membrane, which might be associated with the attenuated increase in serum CK activity and decrease in MVC torque after eccentric exercise. It is necessary to investigate the effects of curcumin on plasma membrane and myofibrils damage in future studies.

The present study found that curcumin ingestion had no positive effects on ROM (Fig. 3), swelling (Fig. 4), DOMS (Fig. 5) and blood markers (IL-6 and TNF-α) of inflammation (Fig. 7). Changes in ROM, upper-arm circumference and DOMS after eccentric exercise were similar to those reported in previous studies (Evans et al. 2002; Nosaka et al. 2006). It was expected that curcumin ingestion would reduce the magnitude of decrease in ROM and increase in upper-arm circumference and DOMS; however, no such effects were found (Figs. 3, 4, 5). Davis et al. (2007) showed curcumin ingestion (10 mg) in mice (considering the body weight difference, it would be 10,000 mg in human) attenuated IL-6 and TNF-α elevation following eccentric exercise. Comparing to this animal study, the amount of curcumin (150 mg × 2) was small in the present study. However, it is known that curcumin is highly lipophilic and poorly bioavailabe, and that more than 8000 mg of curcumin intake did not necessarily increase blood curcumin concentration in human (Kanai et al. 2012). In contrast, the theracurmin that was used in the present study was highly absorbable and more bioavailable when compared with conventional curcumin powder (Sasaki et al. 2011). It is not known how equivalent the amount of theracurmin used in the present study (150 mg × 2) was to that of the previous studies that used conventional curcumin powder (e.g. 8000 mg). The subjects ingested theracurmin before exercise and 12 h after exercise, which resulted in prolonged increases in plasma curcumin concentration above the baseline for 96 h after exercise for the curcumin condition, although the level of plasma curcumin concentration was low (8 < ng/mL) at 48–96 h post-exercise (Fig. 1). Although the range of the subject’s body mass was relatively large (50–91 kg), there was no relationship between plasma curcumin concentration and body mass. A previous study showed that ingestion of theracurmin (150 mg) increased plasma curcumin concentration already at 1 h post-ingestion, and its half-life time was 12 h in healthy adults (Kanai et al. 2012). Thus, it is assumed that the amount of curcumin for each time point (150 mg) was adequate to provide anti-inflammatory effects, if the supplement had such effects. However, the present study found a significant effect of theracurmin supplementation before and after exercise on MVC torque and CK activity only, and no effects on ROM, muscle soreness and swelling. Nosaka et al. (2006) reported that essential amino acid supplementation given 30 min before and immediately after eccentric exercise (total of 7.2 g) did not affect any indicators of muscle damage, but when the supplementation was continued in next 4 days after exercise (morning and evening for ten times over 5 days, total of 36 g) attenuated increase in muscle soreness and ROM. Thus, it is possible that the effects of curcumin supplementation on DOMS and swelling would have been found, if the curcumin had been given on recovery days (e.g. 1–4 days post-exercise). There are many possible supplementation conditions in terms of dose, frequency and time points; a series of studies are warranted to investigate whether a larger dose and more frequent theracurmin supplementation in recovery days after eccentric exercise will provide greater effects on muscle damage.

The polyphenolic component of curcumin exerts anti-inflammatory effects via downregulation of NF-κB, which decreases in the expression of inflammatory cytokines such as IL-6 and TNF-α (Jobin et al. 1999; Shishodia et al. 2007; Davis et al. 2007). Thus, we expected to see smaller increases in IL-6 and TNF-α after eccentric exercise for the curcumin than placebo condition; however, no significant differences between conditions were found, and they did not change significantly for either condition (Fig. 7). Hirose et al. (2004) showed no significant changes in IL-6 and TNF-α at 24–96 h after elbow flexor eccentric exercise and explained that the short exercise time and the small muscle mass involved in the exercise did not affect the inflammatory cytokine levels in the blood. Philippou et al. (2009) reported that knee extensor eccentric contractions increased serum IL-6 concentrations at 6 and 48 h post-exercise, but Miles et al. (2008) showed that IL-6 increased at 8 and 12 h, and returned to the baseline level at 24 h after elbow flexor eccentric exercise. Depner et al. (2010) reported that TNF-α concentration did not change, but IL-6 decreased by 20 % at 1.5 and 4 h after eccentric exercise of the elbow flexors and extensors. Thus, it might be that the present study missed the changes in IL-6 and TNF-α, because no blood samples were taken between immediately and 24 h after exercise. It is also interesting to assess other cytokines (e.g. IL-8, IL-10) and inflammatory markers (e.g. C-reactive protein) in future studies. Previous animal studies have reported that curcumin ingestion attenuated increases in inflammatory cytokine (e.g., IL-6 and TNF-α) in the soleus muscle and oxidative stress markers (e.g., hydrogen peroxide and NADPH-oxidase) in the gastrocnemius muscle at 24 and 48 h after downhill running (Davis et al. 2007; Kawanishi et al. 2013). Therefore, it is also important to examine the effects of curcumin ingestion on inflammatory cytokines and oxidative stress marker in human skeletal muscles.

Many studies that investigated the effect of nutritional supplementation on muscle function have failed to find positive effect on muscle function (Nosaka et al. 2006; Bryer and Goldfarb 2006; O’Fallon et al. 2012). However, the present study demonstrated that highly bioavailable curcumin attenuated the loss of muscle function after eccentric exercise. This may be important for athletes who train and compete regularly, since curcumin ingestion could help them to reduce prolonged loss of muscle function in an intense competition season. However, it should be noted that the present study used untrained men as the subjects and thus the findings of the present study cannot be generalised to other population such as women and trained individuals. Further studies are necessary to test women and trained individuals for the effects of curcumin supplementation on eccentric exercise-induced muscle damage. Furthermore, if curcumin supplementation provides strong anti-inflammatory effects, it should be investigated whether chronic curcumin intake attenuates muscle adaptations induced by resistance training. Trappe et al. (2001) reported that ibuprofen blunted protein synthesis after knee extensor eccentric exercise. Thus, it might be that chronic consumption of curcumin blunts muscle hypertrophy. The chronic supplementation of curcumin warrants further studies.

In conclusion, the present study demonstrated that the decrease in muscle strength and increase in serum CK activity after eccentric exercise were attenuated by highly bioavailable curcumin intake at 1 h before and 12 h after eccentric exercise of the elbow flexors in untrained men. These findings suggest that curcumin intake has some beneficial effects on recovery of eccentric exercise-induced muscle damage.

## Abbreviations

CK: Creatine kinase
DOMS: Delayed onset muscle soreness
IL-6: Interleukin 6
MVC: Maximal voluntary contraction
ROM: Range of motion
TNF-α: Tumor necrosis factor-α
VAS: Visual analog scale
